# Guidelines for Natural Stone Products in Connection with European Standards

**DOI:** 10.3390/ma16216885

**Published:** 2023-10-26

**Authors:** Paweł Strzałkowski, Ekin Köken, Luís Sousa

**Affiliations:** 1 Department of Mining, Faculty of Geoengineering, Mining and Geology, Wroclaw University of Science and Technology, Wybrzeże Wyspiańskiego 27, 50-370 Wrocław, Poland; 2 Nanotechnology Engineering Department, Engineering Faculty, Abdullah Gul University, Kayseri 38100, Turkey; ekin.koken@agu.edu.tr; 3 Department of Geology and Pole of CGeo—Geosciences Center, University of Trás-os-Montes e Alto Douro, 5000-801 Vila Real, Portugal; lsousa@utad.pt

**Keywords:** natural stones, dimension stones, ornamental stones, guidelines, rock properties

## Abstract

The selection of ornamental stones for specific applications requires technical guidance since it should be based on the durability, service life, and aesthetic value of the stones. In most cases, these fundamentals provide quantitative data on the usability and performance of ornamental stones. The present study attempts to put forward a quantitative classification system for natural stone products concerning critical rock properties. For this purpose, fundamental physical and mechanical rock properties are listed based on European standards. Then, minimum limit values are proposed for different applications of natural stone products based on retrospective analyses of numerous ornamental stone applications. The suggested limit values based on several physical and mechanical rock properties can guide relevant engineers to initially consider possible rock types for use as natural stones in a wide range of applications. In this context, it is believed that the present study contributes to the natural stone industry by discussing the minimum limit values for the consideration of a wide range of rock types possibly usable in the dimension stone industry.

## 1. Introduction

Natural stones have been widely used for architecture and construction purposes since ancient times [1,2,3]. In many regions of Europe, they have characterised traditional buildings and historical monuments [4]. Considering historical and cultural aspects, natural stones represent geographical diversity and preserve stone-built heritage [5]. In addition, the proper use of natural stones for historical purposes enriches the aesthetics of cities and preserves the cultural environment [6]. In this context, natural stones have been of considerable importance regarding historical and cultural heritage [2,7,8,9,10].

The development of the construction and building industry is highly associated with the supply of high-quality raw materials. Nevertheless, compared to other commonly used building materials (e.g., concrete, brick, etc.), the efficiency and productivity of ornamental stone utilisation and treatment processes are quite limited [11,12]. The underlying reason for this phenomenon is that the formability of rocks is harder than those of other construction and building materials. In addition, the generation of a large amount of waste in the exploitation and treatment process of dimension stones restricts their usage. It is estimated that during the treatment process of dimension stones, up to 10–35% of the apparent reserve can be wasted. Nevertheless, the suitability of dimension stones for natural stone production is at the level of 5–60%. Considering these facts, it is estimated that the end products account for about 25% of the apparent reserve of the host rock [7,13,14,15].

On the other hand, the durability and attractiveness of ornamental stones make them eligible to be among the best alternatives for designing construction, building and other engineering projects [3,7].

Selecting the appropriate rock type for relative engineering purposes and making an exact decision regarding its usability and employability can be challenging and require effort on the part of homeowners and contractors [3]. For this purpose, developing maintenance-free buildings is highly desirable and, perhaps, an increasingly technical possibility, but hardly an achievable goal. During the design and operation stages in the natural stone industry, the number of maintenance steps can be reduced depending on the quality of raw materials, construction methods, and environmental and in-use conditions of a structure. All these factors affect the maintenance interval and the service life of engineering structures and buildings composed of natural stones [16]. Therefore, building materials should be selected at the design stage with consideration of their technical characteristics. In addition, it is helpful to extend this characterisation with long-term experimentations. It should be mentioned that any rock type undergoes a natural weathering process that decreases its physical and mechanical properties. Over time, natural stones are subjected to various external factors and harsh environmental conditions which can cause them to deteriorate [17,18]. Therefore, the initial rock properties reflect the usability and suitability of natural stones. The appropriate selection of ornamental stones for specific applications is highly demanded across European countries. However, based on critical rock properties, European standards in the dimension stone industry do not define minimum limit values for ornamental stones, resulting in difficulties and errors in designing and implementing natural stone-based engineering projects. Focusing on this phenomenon, this article attempts to provide the minimum requirements for ornamental stones concerning several European standards. In this context, physical and mechanical rock properties are listed based on different applications of natural stone products. Then, the guidelines for several natural stone products are proposed based on retrospective analyses of ornamental stone applications. 

It should be noted that this article might be declared a modest initiation of a scientific discussion on the development of a comprehensive evaluation methodology to classify and assess rock types used in the dimension stone industry.

## 2. Scientific Background

In the literature, one may notice an interchangeable use of the terms: dimension stones, natural stones, and ornamental stones [5,18,19,20]. These terms are best defined by Carvalho et al. [11] and EN 12670 [21]: “Dimension stone” refers to the required shape and size of the final product and it is considered essentially as a critical building and construction material having structural functions. The term “natural stone” is used to valorise a stone as a natural product—in an “as is” state, while “ornamental stones” signifies not only the commercial objectives but also the end use of the raw material. The last definition (i.e., ornamental stone) embraces all types of rocks with varying sizes and shapes. Ornamental stones can range from the small cubes used in street pavements to the thin slate plates used as roof tiles or wall coverings. Additionally, they can be regarded as large blocks exploited for use as coverings and pavement slabs and for use in statuary, funerary art, etc. 

Therefore, ornamental stones seem to be the most pertinent term in the context of the requirements for natural stone products. Given the technical value of stone material, besides their economic value, ornamental stones also have a decorative function [11]. In practice, ornamental stones are selected by individual users based on their subjective aesthetic perceptions (colour, overlap, grain size, etc.). The aesthetics of stones are an integral factor determining their application as building materials with decorative functions. 

The quality requirements concerning the colour of stone are stringent. The colour should be as uniform as possible across the entire deposit. If a stone is classified as a one-coloured type, stripes, inclusions, or veins of a differing colour cannot be accepted for a first-class stone. However, if the stone is classified as multi-coloured, appropriate variation in colours is required. In this case, the colour and structure of the stone must be homogeneous enough so that its market value can be considerable [19]. Although some changes in a stone form an excellent visual pattern and enhance the aesthetic value of the product, these changes can reduce its technical quality [22]. For instance, the uniaxial compression strength (UCS) shows an undulatory variation when the bedding inclination increases [23,24]. Changes in the colour of ornamental stones improve their aesthetic value in many cases; however, they vary according to fashion in decorative construction designs [25]. Qualitatively assessing an ornamental stone, including the aesthetic factor, is difficult. The investigations by Alper Selver et al. [26], Haileslassie et al. [27], and Pereira et al. [28] indicated that image processing techniques could be successfully applied to measure the variations in the surface colour of some ornamental stones. A more comprehensive and universal view of stone aesthetics was conducted by Akkoyun and Faut Toprak [29], who proposed a fuzzy model to classify the quality of dimension stones with consideration of colour properties. Yarahmadi et al. [25] developed a quality coefficient formula that illustrates the homogeneity and aesthetic index of dimension stones.

In the context of stone selection for a specific application, it is highly recommended to carefully consider the characteristics of rocks in a detailed manner. Carvalho et al. [11] emphasised that aesthetics can be considered for the technical evaluation of ornamental stones because they result from the conjoined perception of a set of criteria, namely, the colour, the texture, and the presence of veins and discontinuities. However, technical characteristics from the point of view of practical use become much more relevant here. In addition, the method and way of installing the finished stone products are of significant importance. This is confirmed by the many drawbacks and problems associated with the use of these natural building materials [16,30,31,32,33,34,35]. 

As a natural material, stone can be one of the most difficult building materials to properly evaluate, select, and specify [36]. Aladejare and Wang [37] pointed out that rock properties are fundamental and integral parts of many aspects in engineering and geological practice. Since they are necessary inputs in the analysis of rock engineering problems, these properties are also required for the exploration, planning, and optimal utilisation of rock structures. The design and construction of rock structures are generally influenced by physical and mechanical rock properties and environmental factors. In addition, rock properties are also essential in rock mass classification systems [38,39,40].

Since natural stones are some of the most challenging building materials, whose technical properties highly affect the durability of a structure, the selection of the proper rock type as a natural stone is of prime importance. Considering the physical and mechanical properties of ornamental stones, it is possible to see their strong interdependence [37,41,42,43,44,45,46,47]. Physical properties coupled with the mechanical behaviour of rocks are influenced by the mineralogical composition, degree of cementation, and grain size [7,31,48,49,50,51,52,53]. More profoundly, Aladejare and Wang [37] showed that bulk density, specific gravity, and unit weight generally show low variability, while porosity and water content show high variability in igneous, sedimentary, and metamorphic rocks. Deformation and strength properties are more variable rock properties than typical physical properties. In addition, sedimentary rocks show more variability than other rock types. Nevertheless, the mechanical behaviour and failure modes of volcanic rocks are qualitatively similar to sedimentary rocks [54].

## 3. Standard Requirements for Natural Stone Products

Rocks used as building materials must satisfy high quality standards to ensure the long-term durability of engineering structures [7,42]. They are exposed to external loads and harsh environmental conditions [17,18] in all processes, from production to use in the field. However, natural stone is one of the most durable building materials for road paving, flooring, wall cladding, or decorative architectural purposes [8,55]. 

**Figure 1:**
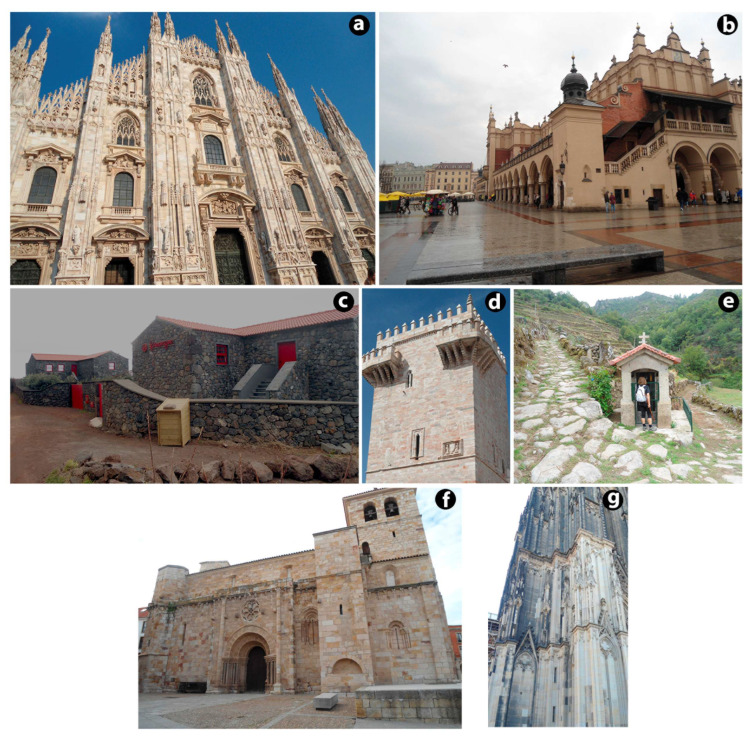
Examples of ornamental stone applications: (**a**) Milan Cathedral, Italy; (**b**) Main Market Square in Krakow, Poland; (**c**) houses, Saint Jorge Island, Portugal; (**d**) Estremoz Castle, Portugal; (**e**) small chapel for worship, Sistelo, Portugal; (**f**) San Juan de Puerta Nueva Church, Zamora, Spain; (**g**) Cologne Cathedral, Germany.

**Figure 2:**
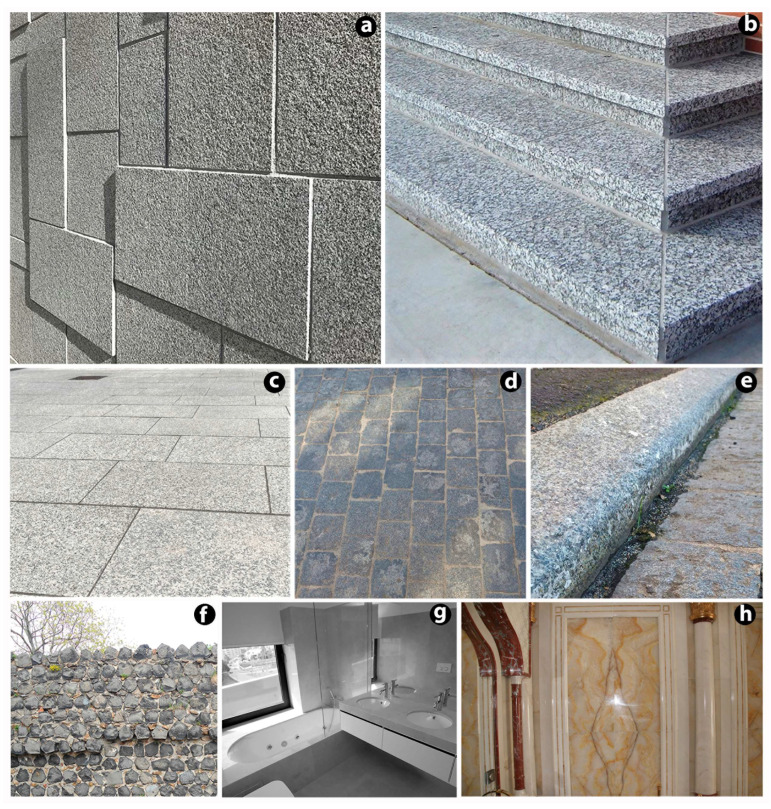
Applications of natural stone products: (**a**) cladding slabs; (**b**) floor slabs, skirting slabs, or stair slabs; (**c**) paving slabs; (**d**) setts; (**e**) kerbs; (**f**) stone masonry; (**g**) bathroom countertop; (**h**) curved elements.

Ornamental stones are widely used in architecture or construction (Figure 1) and, in some cases as an alternative to hardwood, tile and masonry [3]. In particular, ornamental stones are more commonly used for wall and pavement cladding purposes [30]. Cladding slabs (Figure 2a) are thin elements for wall cladding or ceiling finishes fixed to structures mechanically or with mortar or adhesives [56]. Similarly, modular tiles are defined as flat, square, or rectangular natural stone fragments with a thickness of less than 12 mm [57]. A characteristic type of slab is a floor slab, skirting slab, or stair slab (Figure 2b). Each element is a flat fragment of natural stone less than 12 mm thick [58]. Thin natural stone pieces are more sensitive to the external environment and degradation than thick natural stone pieces [4,16]. These stone products are highly prone to external degradation factors. During their service life, stone claddings suffer from continuous degradation that reduces their ability to withstand external surcharge loads. The loss in material quality varies according to the deterioration agents and the nature of the material itself [59].

Stone is widely used for paving and road surfaces in the form of slabs, setts, or kerbs due to its high durability. According to EN 1341 [60], an external paving slab (Figure 2c) is an element used for paving and road finishes in which the working width exceeds up to two times its thickness. On the other hand, a sett (Figure 2d) is a tiny element in which the working width does not exceed two times its thickness and the length does not exceed two times its width [61]. A piece 300 mm in length is commonly used as a kerb [62]. A typical kerb is shown in Figure 2e. The main function of using a kerb is to provide structural support to the edges of roads and channel rainwater away [34]. Stone pavements belong to the broader family of element or segmental pavements, i.e., pavements in which the surface course is made up of individual units placed close to each other and embedded in a bound or unbound bedding layer or laying course [32]. Structural degradation of road pavements is a cause of reduced road safety levels connected to the onset of defects and singularities in correspondence with which the homogeneity and regularity of the pavement are lost. The above-mentioned factors have a negative effect on the functionality and comfort of driving and increase the possibility of accidents [32,35].

Masonry units manufactured from natural stone (Figure 2f) are elements whose main intended uses are common, facing, or exposed masonry units in load-bearing or non-loadbearing building and civil engineering applications. These units are suitable for all forms of coursed or random masonry walling, including single leaves, cavities, partitions, retaining walls, and the external masonry of chimneys [63]. Several types of stone masonry can be distinguished: unreinforced random rubble masonry with mortar, unreinforced shaped stone masonry (cut cubes) with mortar, and reinforced masonry with mortar [64]. For natural stone masonry, as for other natural stone products, it is important to observe their deterioration processes, which have a major impact on their service life. It is also important to observe the structural effects of stone masonry degradation. This has important implications for the safety of masonry structures [65,66].

The other group of natural stone products is dimensional stonework, where stone elements can mostly be used in the building sector. These include curved stones or stone elements with three-dimensional stone shapes, including flat stone elements (Figure 2g,h) which are not used as slabs for cladding or as slabs for floors and stairs, or for furniture (e.g., tables and kitchen tops) [67].

There is no doubt that the uses and applications of stone are much broader. The other most common use of ornamental stones is for kitchen and bathroom countertops. In any urban area, ornamental stones are used for entrances, plinths, outdoor benches, etc. They are also used as basins, altars, and ornate fixtures in churches.

Ornamental stones are used for indoor and outdoor furniture. In addition, ornamental stones are considered ideal materials for fireplaces [3,68].

Regulation (EU) No. 305/2011 of the European Parliament and of the Council of 9 March 2011 laying down harmonised conditions for the marketing of construction products and repealing Council Directive 89/106/EEC (a text with EEA relevance) [69] defines a construction product as any product or kit which is produced and placed on the market for incorporation in a permanent manner in construction works or parts thereof and the performance of which has an effect on the performance of the construction works concerning the basic requirements for construction works. The essential characteristics of construction products are defined in harmonised technical specifications (harmonised standards and European Assessment Documents) in basic requirements for construction works.

Harmonised standards for product requirements and test methods for natural stone are widely used in Europe. The requirement standards for natural stones define the properties and methods for their identification depending on the application. Based on the properties of the natural stone, the manufacturer prepares a declaration of performance when it is placed on the market and marks the product with a CE mark. It must be emphasised that it is the obligation of the manufacturer of natural stone products to prepare a declaration of performance. However, despite this obligation, it is not common practice. This is due to a lack of knowledge or the high cost of performing tests to determine the properties of ornamental stones. Companies operating in the extractive industry of ornamental stones are small and medium-sized enterprises with strong links within their own commercial-activity circles [11]. Thus, it is a major challenge to increase the awareness of producers of the need to conduct such tests, as they can guarantee the quality of building products and safeguard the interests of producers and investors.

Since ornamental stones have a wide range of applications, the current standards only provide guidelines for the general use of these natural materials (Table 1). According to the adopted European standards, there is no classification of stone materials and limit values on natural stones, apart from guidelines on expected failure loads for slabs [60] and kerbs [62]. Only the values of the properties specified by the requirements of the standards which characterise a batch of products intended for sale are declared. Based on the previous experiences of natural stone producers and adopted national standards, natural stone can be addressed to specific usage areas [70]. However, this is decided by the developer or designer of the building project. Due to the lack of well-defined technical criteria, rock types cannot be clearly recognised and addressed to proper usage areas [30].

A different solution for determining the requirements for natural stone products is represented by American standards. These standards refer to the lithological type of rock and define the minimum values of stone properties (Table 2). It seems that this solution is much better for the design of construction investments based on natural stones. However, judging the usefulness of natural stones based on a few parameters seems insufficient, and a catalogue focusing on the physical and mechanical properties of natural stones should be present. Furthermore, it is not possible to implement the requirements of the ASTM standards in European practice. This is due to the fact that the methods of determining rock properties are different from those adopted in European solutions. This is also confirmed by Cárdenes et al. [71], who demonstrated different test methods for determining the technical properties of roofing slate.

## 4. Proposal for Classification and Limit Values of the Physical and Mechanical Properties Depending on the Application of Ornamental Stones with Reference to European Standards

The requirements for the limit values of ornamental stone properties should be defined in the technical specifications of the investment in which the stone product should be used. Since there are no minimum requirements for ornamental stones based on their physical and mechanical properties, in this section, several guidelines for ornamental stones are proposed based on previous experiences of a wide range of natural stone applications.

Different approaches to classifying and assessing the quality and durability of ornamental stones become complicated for different products of building materials. In addition, different test methods based on American and European standards are hardly comparable in assessing the physical and mechanical properties of rocks.

Based on the above explanations, a general quantitative classification for ornamental stones is listed in Table 3. The European standards for determining physical and mechanical rock properties detailed in Table 4 are given in Table 1. For different applications of natural stones, another quantitative classification system is also listed in Table 4. The limit values, shown in Table 3 and Table 4, were developed based on the experience of the article’s authors, as well as a review of the technical specifications of building projects in which natural stone was used (e.g., for cladding or floor slabs). The values were also examined and confirmed by experienced designers. The most important and popular parameters defining the usability of stone are apparent density and open porosity, water absorption at atmospheric pressure, water absorption by capillarity, compressive strength, flexural strength, freeze/thaw resistance, abrasion resistance, and slip resistance. Other parameters are relevant for the specific use of ornamental stones and should be considered individually. It should be added that the parameters listed, apart from slip resistance, mainly depend on a stone’s structure. Slip resistance, on the other hand, is closely related to the stone surface treatment process.

It should be noted that in the quantitative classification for ornamental stones (Table 3) and in the classification system (Table 4), the types of ornamental stones are not mentioned. This is related to the use of the stone; for example, if less durable rocks such as sandstone fulfil the minimum requirements, then highly durable rocks such as granite will also fulfil these criteria. Considering the structure and composition of the stone, as well as its aesthetic value, the minimum requirements are some of the factors which allow the selection of ornamental stones for a specific application. Nonetheless, the characteristics of natural stone will mainly determine the durability and lifetime of the material.

It is essential that the selected stone be sound, durable, and able to withstand external factors during its service life [78]. The quantitative classification system (Table 4) for different applications of natural stones is based on the previous experiences of the authors of this paper, as well as a review of the technical guidelines of various building investment projects. The data presented in Table 3 and Table 4 are based on European standards, which indicate the scope of the research conducted (Table 1).

The authors suggest that extensive research is needed to define the minimum requirements for ornamental stones based on the fundamental rock properties. The proposed classification system defined in Table 4 is more a guideline than a list of mandatory requirements. In fact, many traded ornamental stones have porosity values higher than 11% [47,79,80,81], to mention one of the rock properties.

Stones probably will not meet all the requirements at the same time, i.e., in some cases, a stone material is defined as high quality based on a rock property, but it can be defined as a low-quality material based on the other rock properties. The use of ornamental stones should be associated with the actual critical factors affecting the durability of the stone. For instance, ornamental stones used for cladding purposes should be designed and applied safely where flexural strength values are the vital factors, while ornamental stones used for paving purposes should be resistant to abrasion and fragmentation. Whenever the safety and durability of a structure are more significant, minimum requirements should be taken into account. Furthermore, the public/private sectors should consider the appropriate use of ornamental stones. In outdoor utilisations, ornamental stones with low abrasion resistance should not be used, since, after a short time, the slip risk is increased and the safety of pedestrians is compromised. However, in a private backyard, the same material can be used when the owner conscientiously assumes the risk.

Another topic for discussion is the use of hydrophobic and consolidant products, which can help stabilise the physical–mechanical properties of ornamental stones in the long term. These products can widen the range of applications for different ornamental stones due to the improvement of their physical–mechanical properties. For instance, if the porosity is lowered by using a hydrophobic treatment, the physical properties are improved. On the other hand, the use of consolidants can improve the mechanical properties. It must be emphasised that the selection of hydrophobic and consolidating products must be undertaken carefully because an incorrect choice can lead to higher deterioration of the ornamental stones. Failure to follow this practice is a major engineering error.

## 5. Conclusions

In this study, critical rock properties and their testing methods based on European standards are summarised to assess the quality and durability of ornamental stones. Qualitative evaluation of rock properties is extremely difficult and tricky. The different approaches of developers and designers of building projects indicate the problems of selecting the appropriate stone materials for their applications.

It is believed that the suggested minimum guidelines can guide relevant engineers to initially select possible rock types for use as natural stones in a wide range of applications. The presented quantitative classifications and average limit values of the basic properties of ornamental stones are the beginning of a wide scientific discussion in the development of fundamental criteria to select appropriate stone materials in the natural stone industry.

The main conclusions can be drawn as follows:

- In European conditions, the standards for natural stone requirements do not indicate limit values based on physical and mechanical properties.
- Physical and mechanical properties should be some of the fundamental criteria for the selection of ornamental stones depending on different applications.
- A broader discussion should be initiated on the identification and classification of ornamental stones. The present study, in this context, may initiate a scientific discussion on defining the minimum requirements of ornamental stones based on their physical and mechanical properties. However, additional studies and field experiences are still required to improve the suggested minimum requirements.

Further studies should focus on the following considerations:

- Redesign based on the lithological variances (i.e., igneous, sedimentary, or metamorphic) of rocks.
- Inclusion of the use of hydrophobic and consolidation products to stabilise the physical and mechanical properties of rocks in case of exposure to different environmental factors.

## Figures and Tables

**Table 1:** The requirements, the list of standards defining the test methodologies, and the frequencies of testing for particular groups of natural stone products according to European standards.

Geometric, Physical, and Mechanical Characteristics	Standard							
	EN 1341 [60]	EN 1342 [61]	EN 1343 [62]	EN 1469 [56]	EN 12057 [57]	EN 12058 [58]	EN 12059 [67] ^1^	EN 771-6 [63]
	Slabs of Natural Stone for External Paving	Setts of Natural Stone for Exterior Paving	Kerbs of Natural Stone for Exterior Paving	Natural Stone Products—Slabs for Cladding	Natural Stone Products—Modular Tiles	Natural Stone Products—Slabs for Floors and Stairs	Natural Stone Products—Dimensional Stone Work	Specification for Masonry Units—Part 6: Natural Stone Masonry Units
Geometrical characteristics	EN 13373	EN 13373	EN 13373	EN 13373	EN 13373	EN 13373	EN 13373	EN 13373
	Every production lot	Every production lot	Every production lot	Every production lot	Every production lot	Every production lot	Every production lot	Every production lot
Visual appearance	Visual	Visual	Visual	Visual	Visual	Visual	Visual	EN 772-16
	Every production lot	Every production lot	Every production lot	Every production lot	Every production lot	Every production lot	Every production lot	Every production lot
Petrographic description	EN 12407	EN 12407	EN 12407	EN 12407	EN 12407	EN 12407	EN 12407	EN 12407
	Every ten years	Every ten years	Every ten years	Every ten years	Every ten years	Every ten years	Every ten years	Every ten years
Apparent density and open porosity	EN 1936	EN 1936	EN 1936	EN 1936	EN 1936	EN 1936	EN 1936	EN 1936
	Every two years	Every two years	Every two years	Every two years	Every two years	Every two years	Every two years	Every two years
Water absorption at atmospheric pressure	EN 13755	EN 13755	EN 13755	EN 13755	EN 13755	EN 13755	EN 13755	
	Every two years	Every two years	Every two years	Every two years	Every two years	Every two years	Every two years	
Water absorption by capillarity				EN 1925	EN 1925	EN 1925	EN 1925	EN 772-11
				Every ten years	Every ten years	Every ten years	Every ten years	Every ten years
Reaction to fire				EN 13501-1	EN 13501-1	EN 13501-1	EN 13501-1	EN 13501-1
				Every ten years	Every ten years	Every ten years	Every ten years	Every ten years
Flexural strength	EN 12372		EN 12372	EN 12372	EN 12372	EN 12372	EN 12372 or EN 13161	EN 12372
	Every two years		Every two years	Every two years	Every two years	Every two years	Every two years	Every two years
Resistance to fixings				EN 13364				
				Every ten years				
Compressive strength		EN 1926					EN 1926	EN 772-1
		Every two years					Every two years	Every two years
Freeze/thaw resistance	EN 12371 (flexural strength before and after 56 cycles of freeze/thaw)	EN 12371 (compressive strength before and after 56 cycles of freeze/thaw)	EN 12371 (flexural strength before and after 56 cycles of freeze/thaw)	EN 12371 (flexural strength before and after 14 cycles of freeze/thaw)	EN 12371 (flexural strength before and after 56 cycles of freeze/thaw for flooring and after 14 cycles of freeze/thaw for wall finishes)	EN 12371 (flexural strength before and after 56 cycles of freeze/thaw)	EN 12371 (flexural strength before and after 48 cycles of freeze/thaw for stones with mainly horizontal surfaces and after 12 cycles of freeze/thaw for stones with mainly vertical surfaces)	EN 12371
	Every ten years	Every ten years	Every ten years	Every ten years	Every ten years	Every ten years	Every ten years	Every ten years
Thermal shock resistance				EN 14066	EN 14066	EN 14066	EN 14066	
				Every ten years	Every ten years	Every ten years	Every ten years	
Water vapour permeability				EN ISO 10456 and/or EN ISO 12572	EN ISO 10456 and/or EN ISO 12572	EN ISO 10456 and/or EN ISO 12572		EN ISO 12572
				Every ten years	Every ten years	Every ten years		Upon request
Abrasion resistance	EN 14157	EN 14157			EN 14157 (for tiles for flooring and stairs)	EN 14157 (for slabs for flooring and stairs)		
	Every ten years	Every ten years			Every ten years	Every ten years		
Slip resistance	EN 14231	EN 14231			EN 14231 (for tiles for flooring and stairs)	EN 14231 (excluding skirtings and risers)		
	Every ten years	Every ten years			Every ten years	Every ten years		
Thermal conductivity				EN 1745	EN 1745	EN 1745		EN 1745
				Every ten years	Every ten years	Every ten years		Every ten years
Emission of radioactivity				National implementation instructions	National implementation instructions	National implementation instructions		
				Every ten years	Every ten years	Every ten years		
Other dangerous substances	National implementation instructions	National implementation instructions	National implementation instructions	National implementation instructions	National implementation instructions	National implementation instructions		National implementation instructions
	Every ten years	Every ten years	Every ten years	Every ten years	Every ten years	Every ten years		Every ten years

^1^ This European Standard has the status of a national standard.

**Table 2:** Minimum physical requirements for ornamental stones according to US standards.

Physical Property	Test Method (s)	Standard										
		C 503 [72]		C 568 [73]		C 615 [74]	C 616 [75]		C 1526 [76]		C 1527 [77]	
		Standard Specification for Marble Dimension Stone (Exterior)		Standard Specification for Limestone Dimension Stone		Standard Specification for Granite Dimension Stone	Standard Specification for Quartz-Based Dimension Stone		Standard Specification for Serpentine Dimension Stone		Standard Specification for Travertine Dimension Stone	
		Test Requirements	Classification(s)	Test Requirements	Classification(s)	Test Requirements	Test Requirements	Classification(s)	Test Requirements	Classification(s)	Test Requirements	Classification(s)
Density, min, lb/ft3(kg/m^3^)	C 97	162 (2595)	I—Calcite	110 (1760)	I—Low-density	160 (2560)	125 (2003)	I—Sandstone	160 (2560)	I—Exterior II—Interior	144 (2305)	I—Exterior II—Interior
		175 (2800)	II—Dolomite	135 (2160)	II—Medium-density		150 (2400)	II—Quartzitic sandstone				
		168 (2690)	III—Serpentine	160 (2560)	III—High-density		160 (2560)	III—Quartzite				
		144 (2305)	IV—Travertine									
Absorption by weight, max, %	C 97	0.20	I, II, III, IV	12	I	0.4	8	I	0.2	I	2.5	I, II
				7.5	II		3	II	0.6	II		
				3	III		1	III				
Compressive strength, min, psi (MPa)	C 170	7500 (52)	I, II, III, IV	1800 (12)	I	19000 (131)	4000 (24.6)	I	10,000 (69)	I, II	7500 (52)	I
				4000 (28)	II		10,000 (68.9)	II			5000 (34.5)	II
				8000 (55)	III		20,000 (137.9)	III				
Modulus of rupture, min, psi (MPa)	C 99	1000 (7)	I, II, III, IV	400 (2.9)	I	1500 (10.34)	350 (2.4)	I	1000 (6.9)	I, II	1000 (6.9)	I
				500 (3.4)	II		1000 (6.9)	II			700 (4.8)	II
				1000 (6.9)	III		2000 (13.9)	III				
Abrasion resistance, min, hardness	C 241/C 1353	10	I, II, III, IV	10	I, II, III	25	2	I	10	I, II	10	I, II
							8	II				
							8	III				
Flexural strength, min, psi (MPa)	C 880	1000 (7)	I, II, III, IV	-		1200 (8.27)	-		1000 (6.9)	I, II	1000 (6.9)	I
											700 (4.8)	II

**Table 3:** Classification of physical and mechanical properties of natural stone products.

Physical and Mechanical Characteristics	Classification				
	Very Low	Low	Medium	High	Very High
	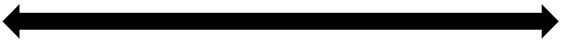				
	Low				High
Apparent density, kg/m^3^	Below 1500.0	1500.0–1800.0	1800.1–2200.0	2200.1–2600.0	Above 2600.0
Open porosity, %	Above 16.0	11.1–16.0	6.1–11.0	1.0–6.0	Below 1.0
Water absorption at atmospheric pressure, %	Above 20.0	14.6–20.0	8.1–14.5	1.0–8.0	Below 1.0
Water absorption by capillarity, g/m^2^·s^0.5^	Above 200.0	150.1–200.0	100.1–150.0	50.0–100.0	Below 50.0
Böhme abrasion value, cm^3^/50 cm^2^	Above 55.0	40.1–55.0	25.1–40.0	10.0–25.0	Below 10.0
Uniaxial compressive strength (UCS), MPa	Below 15.0	15.0–50.0	50.1–120.0	120.1–200.0	Above 200.0
Flexural strength (FS), MPa	Below 4.0	4.0–8.0	8.1–12.0	12.1–16.0	Above 16.0
Freeze/thaw resistance (percentage of UCS/FS before freeze/thaw resistance testing), %	Below 80%USC/FS	80.0–85.0%USC/FS	85.1–90.0%USC/FS	90.1–95.0%USC/FS	Above 95.0%USC/FS

The colours indicate the classification strength of the natural stones.

**Table 4:** Proposal for average limit values of the basic properties of natural stone products depending on the application according to the European standards.

Physical and Mechanical Characteristics	Standard							
	EN 1341 [60]	EN 1342 [61]	EN 1343 [62]	EN 1469 [56]	EN 12057 [57]	EN 12058 [58]	EN 12059 [67]	EN 771-6 [63]
	Slabs of Natural Stone for External Paving	Setts of Natural Stone for External Paving	Kerbs of Natural Stone for External Paving	Natural Stone Products—Slabs for Cladding	Natural Stone Products—Modular Tiles	Natural Stone Products—Slabs for Floors and Stairs	Natural Stone Products—Dimensional Stone Work	Specification for Masonry units—Part 6: Natural Stone Masonry Units
Apparent density, kg/m^3^	≥1800							
Open porosity, %	≤11							
Water absorption at atmospheric pressure, %	≤15							-
Water absorption by capillarity, g/m^2^·s^0.5^	-	-	-	≤150 (when the natural stone product is to be used for elements in contact with a horizontal surface where water may be present)				
Böhme abrasion value, cm^3^/50 cm^2^	≤40		-	-	≤50		-	-
Uniaxial compressive strength (UCS), MPa	-	≥50	-	-	-	-	≥50	
Flexural strength (FS), MPa	≥9	-	≥8.5	≥9				
Freeze/thaw resistance (percentage of UCS before freeze/thaw resistance testing), %	-	≥80% of the UCS	-	-	-	-	≥80% of the UCS	
Freeze/thaw resistance (percentage of FS before freeze/thaw resistance testing), %	≥80 of the FS	-	≥80 of the FS					

## Data Availability

Data sharing is not applicable to this article.
